# Streptococcal Pharyngitis: A Prospective Study of Compliance and Complications

**DOI:** 10.5402/2012/796389

**Published:** 2012-06-21

**Authors:** E. Michael Sarrell, Shmuel M. Giveon

**Affiliations:** ^1^Pediatric and Adolescent Ambulatory Community Clinics-Clalit Health Care Services, 128 Alozorov Street, Tel-Aviv, Israel; ^2^IPROS Network of the Israel Ambulatory Pediatrics Association, Israel Ambulatory Pediatric Association, Tel-Aviv, Israel; ^3^Department of Family Practice, Clalit Health Services HMO, Sharon-Shomron District and Department of Family Practice, Tel-Aviv University, Tel-Aviv, Israel

## Abstract

*Background*. Uncertainty exists concerning the necessity of 10-day antibiotic treatment of group A beta hemolytic streptococcus (GABHS) pharyngitis. *Objective*. To assess the incidence of GABHS recurrence and suppurative and nonsuppurative complications in relation to compliance. *Methods*. (Design). Prospective cohort observational study. (Subjects). 2,000 children aged 6 months to 18 years with sore throat and positive GABHS culture. (Main Outcome Measures). Recurrence of symptomatic culture positive GABHS pharyngitis, incidence of suppurative, and long-term, regional, nonsuppurative complications of GABHS pharyngitis, over a ten year period. *Results*. 213 (11%) of the children received no treatment. Most children received antibiotics for only 4–6 days (in correlation with the duration of fever, which in most cases lasted up to 3 days). Three hundred and six (15.3%) children had clinically diagnosed recurrent tonsillopharyngitis; 236 (12.3%) had positive GABHS findings within 10 to 14 days and thirty-four (1.7%) within 21–30 days after the index positive GABHS culture. The remaining 1.3% had no positive culture despite the clinical findings. Almost all recurrences [236 (11.6%)] occurred within 14 days and 156 (7.6%) in the fully treated group. The presence of fever during the first 3 days of the disease was the most significant predictor for recurrence. Other predictors were the age younger than 6 years and the presence of cervical lymphadenitis. No increase in the incidence of nonsuppurative or suppurative complications was noted during the 10-year follow-up period, compared to the past incidence of those complications in Israel. *Conclusions*. Our data suggests that the majority of children discontinue antibiotics for GABHS tonsillopharyngitis a day or two after the fever subsides. The incidence of complications in our study was not affected by this poor compliance.

## 1. Introduction

Acute pharyngitis is one of the most common infections encountered in primary care clinics. Only 20–30% of patients with group A beta hemolytic streptococcus (GABHS) pharyngitis presents with classical symptoms of the disease [1]. Reliance on clinical judgment alone has a poor predictive value and results in 80% to 95% overestimation of disease [2, 3]. Diagnostic strategies for acute GABHS pharyngitis are thus based on epidemiological factors, signs, and symptoms, as well as the result of throat cultures (TCs) [4]. Several studies have shown that the use of throat culture leads to more judicious use of antibiotics [5–7].

Physicians prescribe antibiotics for acute pharyngitis as they are concerned that patients with this complaint may be suffering from GABHS infection that if left untreated might develop suppurative complications, such as, tonsillar abscess or nonsuppurative complications, such as, rheumatic fever [6, 8]. Antibiotics, however; confer only minor symptomatic benefits for GABHS sore throat. They shorten the duration of symptoms by merely half a day on average [8, 9]. 

Older studies showed that treatment with penicillin reduced the incidence of rheumatic fever. More recent studies have shown that antibiotic use only reduced the incidence of rheumatic fever by a mere 0.5 cases per 100,000 [8]. The importance of preventing rheumatic fever has lessened as the incidence of rheumatic fever and rheumatic heart disease has declined significantly in the last 20 years, from a mean annual incidence of 13.4 per 100,000 to 5 per 100,000. Prevalence has decreased as well from 5.7 per 1,000 in the eighties to 0.5 per 1,000 in 2000 [8, 10, 11]. 

Treatment failure, defined as recurrence of streptococcal pharyngitis, is quite common. This failure probably stems from the fact that about 20% of children with GABHS is infected with bacteria which contain M protein, a virulence factor located on the surface of the bacterial wall that confers resistance to commonly used antibiotics [12]. Newer beta-lactamase-resistant antibiotics did not prevent this treatment failure [13, 14]. 

Review of the literature from 1945 to 1999, which includes 10,484 cases of GABHS sore throat, found that antibiotic treatment reduced the occurrence of acute otitis media, a common complication of this disease, by a mere 25%, compared to the placebo group and sinusitis by only 50% [13]. Rheumatic fever, a nonsuppurative complication, was reduced by less than 33%, compared to placebo [8, 10, 11, 15].

 In addition to the uncertainly in the scientific literature, parents seem to be uncertain regarding the benefits of antibiotic treatment for acute GABHS pharyngitis and tend to stop treatment earlier than prescribed [16]. In a pilot study, we randomly followed 75 children with GABHS pharyngitis for 6 months and have found that more than 75% of them did not complete ten days of antibiotics. This finding led us to conduct a multisite, prospective cohort observational study, the results of which are reported here. The goal of this study was to determine whether noncompliance with antibiotic treatment affects short-term or long-term complications.

## 2. Methods

### 2.1. Study Design

A cohort of 2000 children was followed prospectively for 10 years.

### 2.2. Study Site

Two central, primarily rural, and agricultural regions of the largest Health Maintenance Organization (HMO) in Israel, comprising approximately one million patients.

### 2.3. Patient Selection

**Figure 1:**
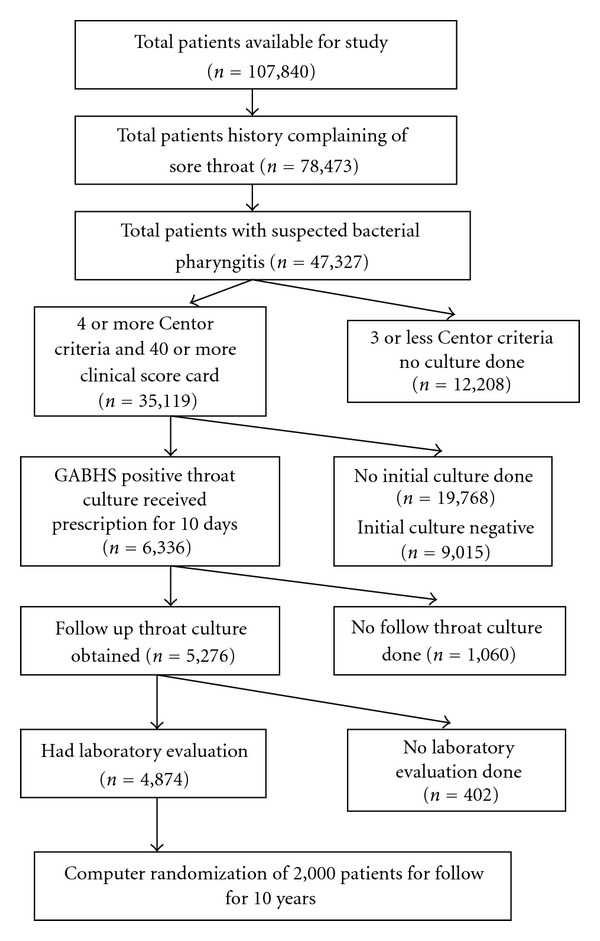


Using a standard protocol, we located from our computerized data base 107,840 patients, aged 6 months to 18 years, who were examined by their primary care physician for upper respiratory tract infection, tonsillitis, pharyngitis, sore throat, tonsillopharyngitis, neck pain, cervical lymphadenopathy, PTA, RPA, from January 1, 1999 until December 31, 2000. We then accessed the charts of 78,473 of these children who were diagnosed with infected throat or one of the differential variants, excluding all children diagnosed as having viral upper respiratory infections. 47,000 of these patients were formally diagnosed with acute pharyngitis or acute tonsillitis and received a prescription for antibiotics, indicating that their physician suspected bacterial disease. In the index visit, 35,000 of these children had at least four out of five symptoms in the modified Centor criteria used for this study and Nadir modified Breese Epidemiological and Clinical Score Card (ECSC) that has 91% sensitivity and 98% specificity when the score was above 15 (score between 4 and 36) for the diagnosis of GABHS [17–19]. The charts of these children were checked to see if throat swabs were taken. These swabs were cultured on standard blood agar plates. Colonies yielding beta-hemolysis were grouped for surface carbohydrate assessment by using a latex bead agglutination test (Figure 1).

Of the 6336 children (with positive cultures with 4 or more Centor criteria and 15 or higher ECSC ), 4,775 parents consented to enroll their children to the study (Figure 1). Excluded from the study were children who were diagnosed as GABHS chronic carriers or who had suffered from post-GABHS complications; had any chronic illness, such as, renal or hepatic impairment; had bleeding disorder; had congenital or acquired immunodeficiency or suffered from malignancy. 

Two thousand of 4,775 consenting families were randomly selected as eligible for followup. Initial patient/parent contact was made by one of the authors (M. Sarrell) within 3 to 5 days of the initial positive throat culture. At that time, initial information regarding the illness and whether a prescription for antibiotics was given by the primary care physician. 

### 2.4. Followup

#### 2.4.1. Physicians

The attending physicians of the two thousand study patients were contacted by email within 48 hours of the enrollment by one author (M. Sarrell). The physicians were requested to inform the authors of any additional cultures taken during therapy, and to request that they obtain two additional throat cultures and engage in improve adherence strategies by providing information, counseling, reminders, reinforcement, and if needed personal attention or supervision. While repeated cultures are not routinely recommended for asymptomatic patients who have completed a course of antimicrobial therapy, in light of the poor compliance with treatment in the pilot study, performance of such follow-up cultures was considered important for the purposes of the study. The first follow-up culture was performed within 10 to 14 days of the initial positive culture regardless of treatment status. The purpose of this culture was verification of antibiotics treatment failure or persistence of GABHS in the oropharynx of the untreated patients. The second additional throat culture was taken between days 21 and 30 after the initial positive culture, regardless of treatment status, to ascertain the presence of residual GABHS or recurrences. Treating physicians were also requested to obtain blood for liver enzymes, renal function tests, and urine analysis from all the participants and to perform annual follow-up evaluation thereafter. 

#### 2.4.2. Patients

A second patient/parent contact was made by our study coordinator within 10 to 14 days of initiation of antibiotic treatment. She collected information about demographic characteristics, past medical history, febrile status, need for repeat throat culture during the treatment period (that was not part of the study protocol), and type of medication prescribed. During this contact she obtained information about the number of days of actual treatment, omission compliance and complications, the patients/parents perceived as deriving from the treatment (or lack thereof). Furthermore, we ascertained the collection of the remaining bottles or tablets of antibiotics.

The computerized charts of the participants were searched within 2 to 4 weeks of the second patient/parent contact for additional information, including demographic characteristics, medical and environmental history, initial clinical data, such as, in-office fever evaluation, results of the physical examination, additional culture taken, type of antibiotics used, and disposition of prescription received. A second search of the computerized medical charts was performed by our research assistant between 30 and 90 days of initiation of medical treatment, to ascertain that the 2 requested throat cultures were obtained. Relapse or recurrence of clinical or bacterial pharyngitis, suppurative or nonsuppurative complication, or even whether the participants complained of any sore throat within 30 days of completion of treatment were also evaluated. In order to assure that all possible short-term complications that occurred within 90 days of the index case were obtained, an additional comprehensive search of the HMO database was done within 120 days of the second computer search. We ascertained that findings that were either not available on the original computerized chart or were seen by other than their primary care physician, (e.g., emergency departments, patients that relocated), were not overlooked.

The charts of the participants were then reviewed by one of the authors on a yearly basis, from January 2000 to January 2010, noting possible late nonsuppurative complications of GABHS infection. No patients were lost to followup, even if they had changed physicians, due to our ability to track them through the centralized database to their new physician or another HMO. The children that were enlisted to the army (and thus not members of any HMO during their military service) were contacted either through their former attending physician or the military physician. 

### 2.5. Outcome Measures

Minor treatment failure was defined as any clinical or bacterial recurrence of pharyngitis during the short-term follow-up period and its correlation to compliance with treatment. 

Major treatment failure was defined as retropharyngeal or peritonsillar abscess or long-term complications, such as rheumatic fever. 

### 2.6. Sample Size

Suppurative complications were chosen as a model, because the nonsuppurative complications (rheumatic heart disease, arthritis, carditis) have been practically eradicated in our region. This cohort study was designed to analyze rare events (according to the CIOMS classification 1–10 events per 10,000 children years). The adverse events of particular interest were PTA and RPA. The annual incidence of peritonsillar abscess (PTA) in our region is 2–4 cases per 100,000 and the incidence of retropharyngeal abscess (RPA) is 5–7 cases per 100,000. 

Power calculations suggested 6,500 to 7,000 person-years of intervention would be needed to detect a 22% difference in PTA and RPA between the fully treated (FT) and partially treated (PT) arms of the study population. Furthermore, one-sided alpha of 0.025, a statistical power of 95%, and the PTA/RPA incidence given above showed that approximately 19,000 children-years would be needed to show the noninferiority of FT versus PT. Since the primary outcome of interest is the PTA/RPA hazard ratio between FT and PT. The null hypothesis to be tested is HR PTA/RPA > 2 (i.e., the PTA/RPA hazard ratio for FT versus PT is higher or equal to 2). The alternative hypothesis is HR PTA/RFA < 2. In a subanalysis the PTA/RPA hazard ratio was calculated for FT versus PT. 

### 2.7. Analysis

For purposes of analysis, participants were divided into four subgroups based on length of treatment: 1st subgroup (untreated), those who did not receive any treatment, 2nd subgroup (partially treated), children that received antibiotics for 1 to 3 days, 3rd subgroup (mostly-treated), children treated for 4 to 6 days, and 4th subgroup (fully-treated) children treated between 7 to 10 days.

Survey responses were analyzed using SPSSWIN, Version 18.0. Data are presented as proportions (with 99% confidence intervals [CIs]), means (with SDs), or medians (with interquartile ranges), using Pearson *χ* ^2^ tests, Student's tests, or Fisher Exact Test. Comparisons of length of treatment according to time and treatment were assessed using the Repeated Measures and Analysis of Variance and the Paired *t* Test. A 2-tailed *P* value of.05 was used to determine the statistical significance of differences observed between groups and to calculate confidence intervals around differences in sample means and odds ratios. We used the McNamara test to measure the changes between the groups and their subgroups regard to the length of antibiotic treatment. 

## 3. Results

Over half of their children (1023, 51%) were between the ages of 6 months and 6.9 years, and over half 1,039 (52%) were female. Most (1524, 76%) lived in a two parent household. The majority of children (1,821, 91%) were prescribed penicillin or amoxicillin, allergic or intolerant to penicillin were treated with cephalosporin 25 (1.5%), erythromycin 109 (5.5%), and azithromycin 45 (2.5%), all medication were prescribed twice daily for 10 days, except azithromycin once daily for 5 days. No statistical correlation was found between the type of antibiotics, the children received, or the demographic characteristics and the complications found in the later medical examinations.

Only 196 children (9.8%) actually completed 10 days of antibiotic treatment. Despite having received a prescription from their physician, two hundred and thirteen participants (11%) did not start taking any treatment whatsoever, including those who did not even purchase antibiotics. As shown in Table 1 a no statistical correlation was found concerning length frequency and duration, palatability, number of daily dose of treatment, but a statistically significant difference was found between all the subgroups concerning the length of antibiotics treatment (*P*   < .0001).

The majority of children (1192, 59.6%) had 3 days or less of fever, defined as any rectal temperature less than 38.5°C or oral temperature less than 37.8°C. The majority of children (1591, 80%) received medication for four to six days at the most (partially treated subgroup). As illustrated in Table 1, the association between the duration of fever and the number of days of treatment was statistically significant (*P* < .0001). 

Of the 306 (15.3%) children with clinically diagnosed recurrent tonsillopharyngitis, only 236 (12.3%) had positive GABHS findings on the throat culture taken within 10 to 14 days after conclusion of the primary infection. An additional thirty-four (1.7%) had a positive second study culture (taken 21–30 days after the index positive GABHS culture). The remaining 46 (1.3%) had a negative culture despite the clinical findings. Of note is the fact that the majority (156, 66%) of the positive study culture at 10–14 days were found among the subgroup treated for 7 to 10 days. No such positive results were found in the subgroup treated for 1 up to 3 days. Furthermore, no positive GABHS throat cultures were found on the second study culture in the untreated group. The majority (26, 76%) of positive GABHS cultures were in the mostly treated subgroups. As illustrated in Table 2, these findings were both statistically significant (*P*  <  .0001) (Table 2). 

Cervical lymphadenitis, acute otitis media, and impetigo were the only suppurative complications noted. Cervical lymphadenitis was the most prevalent short-term complication. 110 (5.5%) children developed cervical lymphadenitis, most (52, 47%) among the 6 to 7 days treatment subgroup and 33 (33%) among the 4 to 5 days treatment subgroup, a significant difference among the treatment subgroups (*P* < .0001). None of the children participating in this study developed other suppurative complication. Additionally, no children developed nonsuppurative complications during 10 years of followup nor did any of the children develop IgA nephropathy during the follow-up period. Furthermore, they were no association between the five modified Centor criteria and development of complication, even when stratified by type of antibiotics or the season of the year.

Altogether, 304 (15%) new onset cases of acute otitis media (AOM) were diagnosed within 30 days of the initial diagnosis. However, only 31 (10%) of those were in the untreated subgroup, as compared to 141 (46%) in the 7 to 10 days treatment subgroup, 98 (38%) in the 4 to 6 days treatment subgroup and 98 (33%) in the 1 to 3 days subgroup, a statistically significant difference among the all treatment subgroups (*P*  <  .0001). 

Attempting to elucidate the possible causes for the differences between the recurrence of GABHS and the length of antibiotic treatment or clinical score on enrolment or illness severity, a multivariate stepwise logistic analysis was performed. The duration of fever was the most significant predictor for such recurrence, age under 6 years being less significant, while treatment for 7 to 10 days had no significant influence on the recurrence of GABHS. 

Of note is that the contribution of the duration of fever was apparent even after controlling for the concomitant influence of age, gender, medical history, single- or two-parent home, type of antibiotics or the season of the year, and concomitant illnesses, such as, conjunctivitis, otitis media, upper respiratory infection, gastroenteritis, or lymphadenitis, which may have otherwise explained the influence of the length of treatment in relation to those illnesses (Table 3).

## 4. Discussion

This study found a very poor parent/child compliance to antibiotic treatment prescribed for symptomatic, culture-positive GABHS tonsillopharyngitis. 11% of children did not start taking any treatment at all, and only 10% completed a full course of treatment. The reason for this low rate of compliance is unclear, but it coincides with other reported studies [14, 16]. We speculate that a large proportion of lack of compliance is the parent's sensation that antibiotics are potentially dangerous [17] and overprescribed [18, 19]. Despite this poor compliance, in our study as in others [20], there was a very low rate of suppurative complications.

Furthermore, despite the poor compliance, we found no increase in the incidence of acute rheumatic fever, the most dreaded complication of GABHS, in our patients. In fact, since the year 2000, the incidence of RF in our region has declined from 2.2 per 100,000 to 0.2 per 100,000 in 2008, according to the epidemiological department of the Israeli Ministry of Health. This is in concordance with other developed countries, including, the United States, where the original recommendation for 10 days of antibiotic treatment of GABHS originated. In that country, the incidence of RF has declined steadily since the 1950s. Currently there are only 10 cases of RF per 100,000 patients with GABHS pharyngitis, and only 1 case per 10,000 patients with acute rheumatic fever develop rheumatic heart disease [20–22]. In fact, concomitant with the increased use of antibiotics, recurrence of GABHS in the USA rose from 9% and 10.7% in the years 1975 to 1979, respectively, to 25.9% and 37.5% in the years 1995 to 1996, despite the decline in acute rheumatic fever [22].

14% of patients in our study had a recurrent infection, proven by a GABHS-positive throat cultures. This percentage is similar to other studies which found that penicillin failed to eradicate GABHS from the throat in approximately 13% to 26% of the patients evaluated [23, 24].

The majority of recurrences in our study were in the younger group (mean age of 10.2 years, and 60% of them younger than 9.9 years). This is consistent with other published studies where such recurrences were more frequent among children aged 1 to 8 years than among children aged between 13 and 19 years [24, 25]. 

Historically, prescribing 10 days of oral penicillin began in the 1950s, substituting the intramuscular injections of long-acting parenteral penicillin, based on surrogate markers of eradication of GABHS from the tonsillopharynx. However, no study has conclusively proven that this practice unequivocally prevents acute rheumatic fever [26, 27]. Even though orally prescribed penicillin appeared to be equally effective for clinical and laboratory resolution of signs and symptoms, it is difficult to administer and expensive, considering the staggering financial burden of approximately 140 office visits per annum per 1,000 children younger than 15 years [28, 29]. Substituting azithromycin or cephalosporins for penicillin was found to produce better bacteriological and clinical results and also required a shorter course of treatment [30, 31]. 

### 4.1. Limitations of the Study

 This study examined healthy children. Our results may not apply to adults, sick people, or chronic GABHS carriers. It does not address the optimal length of treatment required to achieve appropriate eradication of the microbe or whether complete eradication is required at all. The method of pill/doses counts is not a good measure of adherence, but due to it simplicity and empiric nature it was found adequate for this study. We were unable to assess the true variation of the incidence of acute rheumatic fever, due to the fact that this disease has been practically eradicated from our population. 

## 5. Conclusion 

Our data suggest that the large majority of parents/patients stop administering antibiotics to their children who suffer from GABHS prior to the completion of the recommended course. It appears that they cease as soon as the symptoms subside. This “incorrect” use appears to have no apparent negative consequences. 

We believe that the frequency and length of treatment with antibiotics can be reduced. A more judicious use of antibiotics would promote and improve compliance, cut costs, and prove more convenient to parents and children alike.

## Figures and Tables

**Table 1:** Patient demographic characteristics in subgroups; age, medical-associated illnesses, and antibiotics prescribed at initial presentation. (% in parenthesis).

Length of treatment in subgroup	No treatment *n* = 213 (11)	1 to 3 days *n* = 979 (49)	4 to 6 days *n* = 612 (30)	7 to 10 days *n* = 196 (10)	*P* value
Mean age of patient enrolled					

Mean Age	9.2	10	11	11	.04

Patient demographic by group					

Age 0.5 to 5.9 years *n* = 1023 (51)	137 (13)	469 (46)	326 (32)	91 (9)	<.0001
Age 6 to 9.9 years *n* = 499 (25)	36 (7.5)	245 (49)	145 (29)	73 (14.5)	<.0001
Age 10 to 13.9 years *n* = 378 (19)	40 (10)	195 (52)	116 (31)	27 (7)	<.0001
Age 14 to 18 years *n* = 100 (5)	0	70 (70)	25 (25)	5 (5)	<.0001

Febrile days					

1 or less days of fever *n* = 1716 (85)	175 (10)	881 (51.5)	470 (27.5)	190 (11)	<.0001
1 to 3 days of fever *n* = 164 (9)	35 (21)	27 (17)	97 (59)	5 (3)	<.0001
4 to 6 days of fever *n* = 120 (6)	3 (2.5)	71 (59)	45 (37.5)	1 (1)	<.0001

Medically associated illnesses					

Conjunctivitis *n* = 36 (1.5)	5 (14)	8 (22)	9 (25)	14 (39)	<.0001
Otitis media *n* = 39 (1.5)	3 (8)	9 (23)	15 (38)	12 (31)	<.0001
URI *n* = 74 (4)	7 (10)	34 (46)	23 (31)	23 (13)	<.0001
Gastroenteritis *n* = 25 (1)	8 (32)	12 (48)	5 (20)	0	<.0001
Lymphadenitis *n* = 1831 (92)	167 (9)	925 (51)	553 (30)	186 (10)	<.0001

Antibiotics prescribed					

Penicillin *n* = 1210 (60.5)	60 (5)	533 (44)	476 (39)	141 (12)	<.0001
Amoxicillin *n* = 611 (30)	105 (17)	361 (59)	111 (18)	34 (6)	<.0001
Azithromycin *n* = 109 (5.5)	37 (34)	52 (48)	20 (18)	0	<.0001
Cephalosporin = cefovit *n* = 25 (1.5)	7 (28)	17 (68)	0	1 (4)	<.0001
Erythromycin *n* = 45 (2.5)	4 (9)	16 (36)	5 (11)	20 (44)	<.0001

**Table 2:** Medical and laboratory *recurrences* of group A beta-hemolytic streptococcal tonsil opharyngitis and short-term complications (%).

Length of treatment in subgroup *n* = 2000 (100)	No treatment *n* = 213 (11)	1 to 3 days *n* = 979 (49)	4 to 6 days *n* = 612 (30)	7 to 10 days *n* = 196 (10)	*P* value
Medical presentation					

Recurrence symptomatic pharyngitis within 30 days of initial culture *n* = 306 (15.3)	44 (2.2)	88 (4.3)	166 (8.4)	8 (0.4)	<.0001

Laboratory culture positive					

Recurrence of pharyngitis with positive GABHS throat culture within 10 to 14 days *n* = 236 (12.3)	40 (2.1)	0	40 (2.1)	156 (8.1)	<.0001
Recurrence Pharyngitis with positive GABHS throat culture within 21 to 30 days *n* = 34 (1.7)	0	4 (0.2)	26 (1.3)	4 (0.2)	<.0001

Suppurative complication					

Cervical lymphadenitis *n* = 110 (5.5)	10 (0.5)	33 (1.7)	52 (2.5)	15 (0.8)	<.0001
Otitis media *n* = 304 (15.2)	31 (1.6)	98 (4.9)	141 (7)	34 (1.7)	<.0001
Impetigo *n* = 3 (0.15%)				3 (0.15)	

**Table 3:** Stepwise linear regression model of the modification of the days of treatment with antibiotics against possible explanatory variables.

Independent variables (2)	Recurrent positive GABHS throat culture within 7 days			Recurrent positive GABHS culture within 8 to 30 days			Recurrent clinically diagnosed tonsillopharyngitis		
	*b*	Significance	*R* ^2^	*b*	Significance	*R* ^2^	*b*	Significance	*R* ^2^
Constant	−19.437	0.994		−23.082			−20.168	0.000	
Treatment days	0.49	0.000	0.392			0.06		0.000	0.093
1 to 3 days	−2.47.	0.000		−2.950	0.999		−4.742	0.000	
4 to 6 days	−33.35	0.983		2.120	0.999		−1.673	0.000	
7 to 10 days	−0.347	0.432		34.130	0.991		−0.908	0.244	
Age		0.000	0.185					0.002	0.016
0.5 to 6.9 years	3.515	**0.015** ^ ∗^		2.997	**0.011** ^ ∗^		1.288		
7 to 13.9 years	−32.589	0.986		3.542	0.998		2.418		
Older than 14 years	2.098	0.000		0.87	1.000		1.389		
Neonatal period	17.611	0.985	0.027	−31.747	0.994		−18.332	0.996	0.000
Sex	1.098	0.005	0.006	−32.139	0.983		−1.991	0.000	0.016
Familial status	17.443	0.994	0.009	(1)			18.005	0.995	0.008
Febrile days		1.000	0.015		1.000	0.303		0.000	0.284
1 to 3 days	6.068	**0.02** ^ ∗^		19.421	0.980		5.428	0.000	
4 to 6 days	−33.350			30.993	0.997		−18.318	0.998	
Clinical score	0.37			(1)		0.21			
Lower than 15	12.69	0.67	0.002	3.652	0.971	0.53	5.213	0.872	
Higher than 15	14.32	0.21	0.07	17.23	0.823		2.314	0.651	
Associated illness	(1)				0.986	0.065		0.078	0.223
Otitis media	(1)				0.997		−1901	0.127	
Conjunctivitis	(1)				0.978		25.743	0.997	
URI	(1)				0.548		−1.008	0.209	
Lymphadenitis	3.678	**0.013** ^ ∗^			1.000		2.672	0.019	
Variables not in the model			0.001			0			0

Total *R* ^2^			0.635			0.631			0.645

^∗^ Statistically significant.

Notes:

(1) Variables rejected from the model due to lack of significance (*P* < .05).

(2) Dependant variable according to two definitions (i) the difference in antibiotics treatment days (ii) the variable that influences the recurrence of streptococcal pharyngitis.

(3) First group (dummy variable): positive throat culture up to 14 days = 1 treatment days, age, sex, marital status, neonatal complication, associated illness, and fever = 0.

(4) Second group (dummy variable): positive throat culture over 14 days = 1, treatment days, age, sex, marital status, neonatal complication, associated illness, and fever = 0.

(5) Treatment days (dummy variable): up no treatment = 1, 1 to 3 days, 4 to 6 days and 7 to10 days of treatment = 0.

(6) Age (dummy variable): until 5.9 years = 1, 6 to 9.9 years, 10–13.9 years, and 14–18 years = 0.

(7) Sex (dummy variable): male = 1 female = 0.

(8) Familial status (dummy variable): single-parent household = 1, two-parent household = 0.

(9) Neonatal period (dummy variable): no complication = 1, complication = 0.

(10) Associated medical signs and illness (dummy variable): cervical lymphadenitis = 1, conjunctivitis, AOM, URI, gastroenteritis = 0.

(8) Days with fever (dummy variable): *no fever* = 1, 1 to 3 days, and 4 to 6 days = 0.

(9) Clinical score card (dummy variable): CSC lower than 15 = 0 CSC higher than 15 = 1.

## Abbreviations

GABHS: group A beta hemolytic streptococcus
TC: throat culture
PTA: Peritonsillar abscess
RPA: Retropharyngeal abscess
HMO: Health Maintenance Organization.

## References

[1] Little PS, Williamson I (1994). Are antibiotics appropriate for sore throats? Costs outweigh the benefits. *British Medical Journal*.

[2] Pichichero ME (1995). Group A streptococcal tonsillopharyngitis: cost-effective diagnosis and treatment. *Annals of Emergency Medicine*.

[3] Lin MH, Fong WK, Chang PF, Yen CW, Hung KL, Lin SJ (2003). Predictive value of clinical features in differentiating group A *β*hemolytic streptococcal pharyngitis in children. *Journal of Microbiology, Immunology and Infection*.

[4] O'Brien KL, Schwartz B, Facklam R Population based active surveillance for invasive group A Streptococcus.

[5] Pichichero ME (1998). Group A beta-hemolytic streptococcal infections. *American Academy of Pediatrics*.

[6] Robertson KA, Volmink JA, Mayosi BM (2005). Antibiotics for the primary prevention of acute rheumatic fever: a meta-analysis. *BMC Cardiovascular Disorders*.

[7] Del Mar CB, Glasziou PP, Spinks AB (2006). Antibiotics for sore throat. *Cochrane Database of Systematic Reviews*.

[8] Del Mar CB, Glasziou PP, Spinks AB, Saliba WR, Mader R (2000). Antibiotics for sore throat. *Israel Medical Association Journal*.

[9] O'Brien KL, Schwartz B, Facklam R Population based active surveillance for invasive group A Streptococcus.

[10] Olivier C (2000). Rheumatic fever—is it still a problem?. *Journal of Antimicrobial Chemotherapy*.

[11] Habib GS (1997). *Rheumatic Fever in the Nazareth Area During the Last Decade*.

[12] Bidet P, Plainvert C, Doit C (2010). Streptococcus pyogenes or groupA streptococcal infections in child: french national reference center data. *Archives de Pediatrie*.

[13] Schaad UB (2004). Acute streptococcal tonsillopharyngitis: a review of clinical efficacy and bacteriological eradication. *Journal of International Medical Research*.

[14] Bisano AL, Gerber MA, Gwalenty JM, Kaplan EL, Schwartz RH (2002). Practice guidelines for the diagnosis and management of group A streptococcal pharyngitis. Infectious Disease Society of America. *Clinical Infectious Diseases*.

[15] Jacobs MR (2008). Antimicrobial-resistant Streptococcus pneumoniae: trends and management. *Expert Review of Anti-Infective Therapy*.

[16] Llor C, Sierra N, Hernández S (2009). Compliance rate of antibiotic therapy in patients with acute pharyngitis is very low, mainly when thrice-daily antibiotics are given. *Revista Espanola de Quimioterapia*.

[17] Attia MW, Zaoutis T, Klein JD, Meier FA (2001). Performance of a predictive model for streptococcal pharyngitis in children. *Archives of Pediatrics and Adolescent Medicine*.

[18] Breese BB (1977). A simple scorecard for the tentative diagnosis of streptococcal pharyngitis. *American Journal of Diseases of Children*.

[19] Nandi S, Kumar R, Ray P, Vohra H, Ganguly NK (2002). Clinical score card for diagnosis of group A streptococcal sore throat. *Indian Journal of Pediatrics*.

[20] Hofer C, Binns HJ, Tanz RR (1997). Strategies for managing group A streptococcal pharyngitis: a survey of board-certified pediatricians. *Archives of Pediatrics and Adolescent Medicine*.

[21] Rennie RA (1998). Prospective study of antibiotic prescribed for children. *Canadian Family Physician*.

[22] Centor RM (2009). Expand the pharyngitis paradigm for adolescents and young adults. *Annals of Internal Medicine*.

[23] Vinker S, Zohar E, Hoffman R, Elhayany A (2010). Incidence and clinical manifestations of rheumatic fever: a 6 year community-Based survey. *Israel Medical Association Journal*.

[24] Pichichero ME, Casey JR, Mayes T (2000). Penicillin failure in streptococcal tonsillopharyngitis: causes and remedies. *Pediatric Infectious Disease Journal*.

[25] Kafetzis DA, Liapi G, Tsolia M (2004). Failure to eradicate Group A *β*-haemolytic streptococci (GABHS) from the upper respiratory tract after antibiotic treatment. *International Journal of Antimicrobial Agents*.

[26] Pichichero ME, Green JL, Francis AB (1998). Recurrent group A streptococcal tonsillopharyngitis. *Pediatric Infectious Disease Journal*.

[27] Gerber MA, Tanz RR, Kabat W (1999). Potential mechanisms for failure to eradicate group A streptococci from the pharynx. *Pediatrics*.

[28] Liñares J, Ardanuy C, Pallares R, Fenoll A (2010). Changes in antimicrobial resistance, serotypes and genotypes in Streptococcus pneumoniae over a 30-year period. *Clinical Microbiology and Infection*.

[29] McCaig LF, Besser RE, Hughes JM (2002). Trends in antimicrobial prescribing rates for children and adolescents. *Journal of the American Medical Association*.

[30] Adam D, Scholz H, Helmerking M (2000). Short-course antibiotic treatment of 4782 culture-proven cases of group A streptococcal tonsillopharyngitis and incidence of poststreptococcal sequelae. *Journal of Infectious Diseases*.

[31] Koga T, Rikimaru T, Tokunaga N (2011). Evaluation of short-term clinical efficacy of 3-day therapy with azithromycin in comparison with 5-day cefcapene-pivoxyl for acute streptococcal tonsillopharyngitis in primary care. *Journal of Infection and Chemotherapy*.

